# Transient targeting of the pancreatic cancer stroma as a ‘fine-tuned’ anti-tumor and anti-metastatic therapy

**DOI:** 10.18632/oncotarget.21468

**Published:** 2017-10-03

**Authors:** Claire Vennin, Thomas R. Cox, Marina Pajic, Paul Timpson

**Affiliations:** Paul Timpson and Marina Pajic: The Garvan Institute of Medical Research, Darlinghurst, Sydney, NSW & The Kinghorn Cancer Centre, Cancer Division, Australia and St Vincent’s Clinical School, Faculty of Medicine, University of NSW, Australia

**Keywords:** pancreatic cancer, extracellular matrix, tissue stiffness, Rho-kinase, stromal targeting

Pancreatic cancer is one of the deadliest types of cancer, and despite extensive basic and pre-clinical research efforts, existing therapeutics remain largely ineffective. During pancreatic cancer progression, neo-plastic transformation of epithelial cells is typically accompanied by a fibrotic response, driven by activation of stromal cells, and which leads to extensive deposition and remodeling of the pancreatic extracellular matrix (ECM) and to reduced tumor vasculature patency [1]. The resulting dense and stiff matrix in turn provides cancer cells with a protective niche, activates potent intracellular signaling pathways, and promotes cell proliferation and invasion while also rendering cancer cells less sensitive to chemotherapies. Consequently, targeting the ECM is considered a valid approach to disrupt pancreatic cancer; however previous attempts to use anti-ECM agents have revealed that the pancreatic tumor ECM can both facilitate and restrain cancer progression, suggesting that there is a delicate balance between the anti- and pro-tumor functions of the ECM.

**Figure 1:**
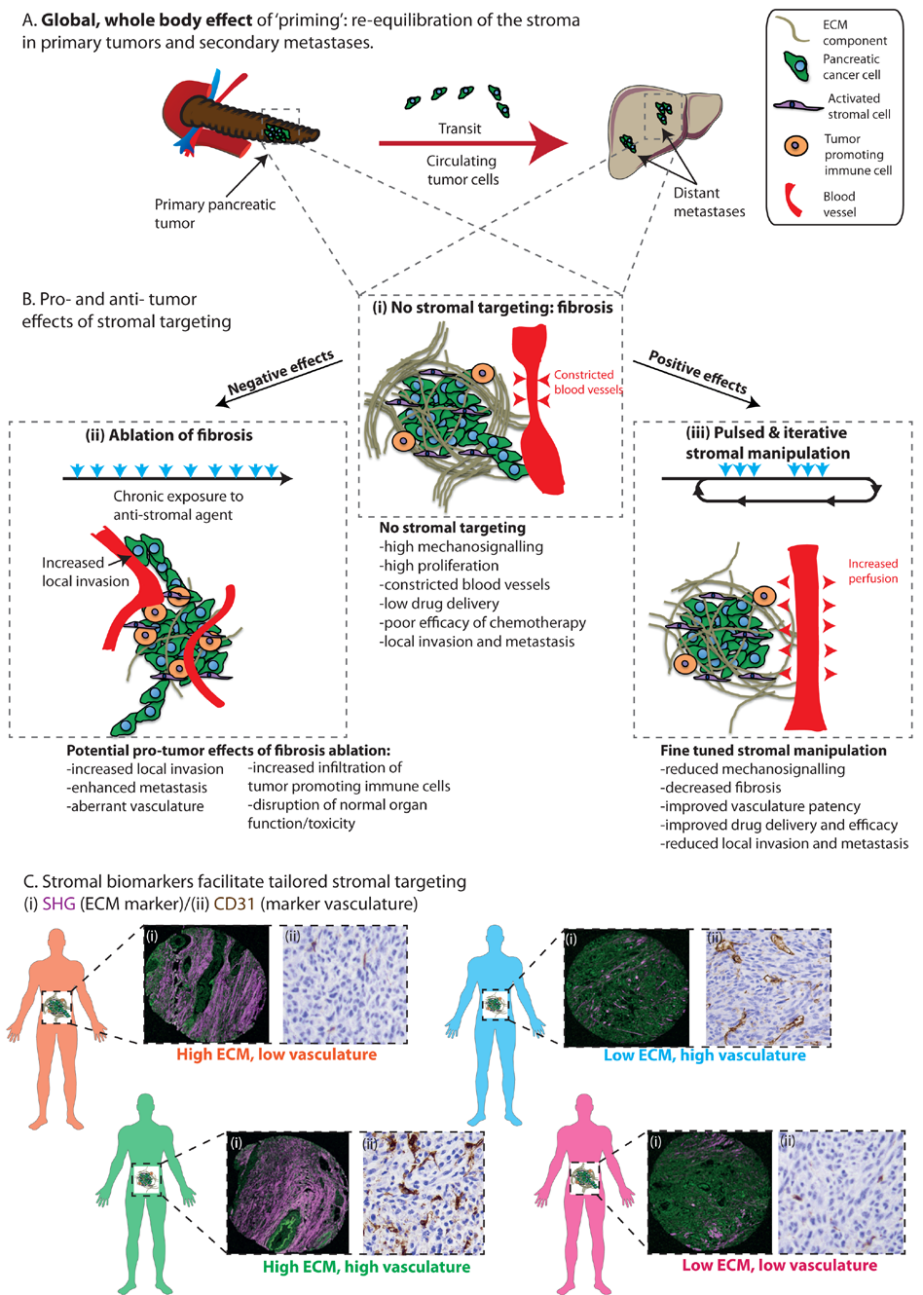
Pulsed and iterative administration of ‘priming’ agents re-equilibrates the stromal balance in primary and secondary sites **A.** ‘Priming’ with anti-fibrotic agents re-equilibrates the stromal balance in both primary and secondary sites, while also impairing cancer cell survival in the circulation. **B.** (i) Stromal features of pancreatic tumors in the absence of stromal targeting and upon (ii) ablation of fibrosis or (iii) pulsed and iterative manipulation of the stroma. **C.** Proposed combination of stromal companion biomarkers (ECM: SHG; vasculature: CD31) for tailored stromal therapies in pancreatic cancer. Adapted from (Vennin et al., 2017).

Recently, we used multi-photon intravital (*in vivo*) imaging technologies and high-end mouse and patient-derived models of pancreatic cancer to uncover a novel therapeutic approach, whereby transient or pulsed ‘priming’ of the tumor using Fasudil, a pharmacological Rho-kinase inhibitor, leads to a decrease in ECM deposition and remodeling and tips the balance in favor of the anti-tumor properties of the matrix while maintaining normal tissue functions of the organ [2]. As such, the administration of Fasudil as a ‘priming’ agent, rather than chronic treatment, led to improved chemotherapy response in primary and secondary sites; while also reducing metastasis to the liver, and prolonging survival in mice bearing patient-derived orthotopic xenografts (Figure 1A). The ‘priming’ regimen triggered decreased remodeling of the ECM, improved vasculature patency, and subsequently has a dual role in drug targeting (Figure 1B, compare (i) and (iii)). In addition, the altered ECM decreased mechano-signaling mediated through integrin pathways and thus rendered pancreatic cancer cells more sensitive to chemotherapy, likely through a reduction of pro-survival and growth cues provided by the fibrotic pancreatic ECM.

Assessment of micro-metastatic events in secondary sites such as the liver revealed that ‘priming’ with Fasudil also impaired cancer cells resistance to shear stress in the circulation, reduced cell streaming and coordinated colonization of the liver, while also blocking ECM remodeling, which is typically required to form a favorable niche at the secondary site (Figure 1A). Together, our work suggests that administration of a ROCK inhibitor as a ‘priming’ agent may be beneficial in the adjuvant and possibly neo-adjuvant settings in pancreatic cancer [2, 3]. This may prove to be a significant advance in the field of stromal targeting in pancreatic cancer, as previous studies have demonstrated that complete ablation of fibrosis in mouse models of pancreatic cancer, for instance via targeting activated α-smooth muscle actin fibroblasts [4] or silencing of the pro-fibrotic Sonic Hedgehog signaling pathway [5] enhances metastasis, increases infiltration of cancer promoting immune cells to the tumor site, and in turn reduces survival in mice (Figure 1B, compare (i) and (ii)). Here, we propose that transient, pulsed administration of a ‘priming’ agent, rather than chronic exposure to anti-ECM drugs, leads to a reduction of the pro-tumor effects of fibrosis and in turn re-equilibrates the pancreatic stroma (Figure 1B compare (ii) and (iii) [2]). While our work demonstrates that ROCK inhibitors can be used as priming agents, this approach could also be applied to recently identified ECM-targets such as hyaluronic acid (HA, [6]) or lysyl oxidase [7].

The dual effect of Fasudil ‘priming’ on both the ECM and tumor vasculature highlights the intricate crosstalk between distinct stromal compartments (Figure 1B). This is in line with recent studies in pancreatic cancer and other types of solid tumors demonstrating a dynamic interplay between various stromal entities such as the ECM, vasculature and immune system within tumor tissues. This body of work emphasizes that targeting of a specific stromal entity potentially alters interconnected stromal compartments and consequently, assessing the subtle downstream effects of stromal targeting is of utmost importance when designing novel stromal-based therapies in order to avoid adverse outcomes (Figure 1B). This will also be particularly relevant for future development of immunotherapy in pancreatic cancer [8], since important remodeling of both the immune landscape and the ECM is known to occur in this disease. Given that the properties of the stroma have been reported to influence T-cell infiltration and activation; combinations of anti-ECM agents using a ‘priming’ regimen, along with immune based therapies may be beneficial in pancreatic cancer.

Pancreatic cancer is a highly heterogeneous disease, and development of patient-centered, targeted therapeutics is emerging as a promising approach. In our study, assessment of ‘priming’ with Fasudil in stratified patient-derived xenografts demonstrated that the level of response to transient ROCK targeting is intimately linked to the initial tumor ECM signature (Figure 1C, [2]). As such, in tumors with a high initial ECM content, ‘priming’ with Fasudil increased response to chemotherapy (gemcitabine/Abraxane) in cancer cells by 50% and almost doubled survival of mice bearing patient-derived pancreatic tumors; while there was only a modest benefit of Fasudil ‘priming’ in tumors with a low ECM content [2]. This suggests that the ECM signature of a tumor could be used as a companion biomarker to identify patients that may benefit from ‘priming’ prior to chemotherapy. Furthermore, given the dual effects of Fasudil on both the ECM and tumor vasculature, a combination of markers covering both stromal compartments (ECM and vasculature) might render patient selection for ‘priming’ more powerful (Figure 1C). This concept aligns with current clinical assessment of targeting of HA using PEGPH20 in pancreatic cancer patients stratified based on the amount of initial HA in their tumor (NCT02715804). In addition, novel platforms are being generated to test pre-clinical therapeutics for precision medicine, such as patient-derived organoids or patient-personalized 3D organotypic matrices [2]. Such models recapitulate many of the features of a patient’s tumor, including the stroma, and also represent cost-effective, high-throughput tools for screening of anti-stromal agents for patient-centered stromal therapies.

In conclusion, chronic treatment regimes are often administered at limited tolerable doses and lead to difficult to manage side effects. Moving forward, researchers and clinicians will have to rethink treatment timeline and dosages; we suggest that iterative ‘priming’ to re-equilibrate the tumor-stroma feedback loop may be of great benefits for pancreatic cancer care.
